# Multimodal Imaging Techniques in Monitoring a Patient with Ocular Decompression Retinopathy

**DOI:** 10.3390/diagnostics13121992

**Published:** 2023-06-07

**Authors:** Justyna Mędrzycka, Paulina Szabelska, Magdalena Rerych, Radosław Różycki, Joanna Gołębiewska

**Affiliations:** Department of Ophthalmology, Military Institute of Aviation Medicine, 01-755 Warsaw, Poland; jmedrzycka@wiml.waw.pl (J.M.); mrerych@wiml.waw.pl (M.R.); rrozycki@wiml.waw.pl (R.R.); joanna.golebiewska@wp.pl (J.G.)

**Keywords:** decompression retinopathy, retinal hemorrhage, intraocular pressure, IOP, acute filtration angle closure, optical coherence tomography, OCT, fluorescein angiography, FA, multimodal imaging

## Abstract

Ocular decompression retinopathy (ODR) is characterized by multiple retinal hemorrhages. It is a rare complication associated with rapid decrease of intraocular pressure (IOP). The course of ODR is usually asymptomatic and self-limiting, which was confirmed by the observation of our patient. In this study, we present a 5-month follow up of a 77-year-old woman with acute right eye (RE) filtration angle closure who developed symptoms of ODR. Clinical examination and multimodal imaging modalities, including color fundus photography, optical coherence tomography (OCT), OCT angiography (OCTA) and fluorescein angiography (FA), were used to confirm the diagnosis and performed regularly in monitoring the course of the disease. Fundus lesions in the RE included diffuse intraretinal hemorrhages in the posterior pole, which gradually resolved during follow-up time. The fundus of the left eye (LE) was normal. The patient underwent conservative therapy, laser therapy and surgery, achieving stabilization of the IOP and improvement of the local condition in the RE. Of the various multimodal imaging techniques, color fundus photography and OCT seemed to be the most specific and helpful in monitoring the patient with ODR.

A 77-year-old female presented to the ophthalmology emergency room in September 2022 due to a severe headache in the right frontotemporal region that had persisted for 2 days. She reported deterioration of visual acuity (VA) in the right eye (RE) accompanying nausea and vomiting. The previous day, the patient had been consulted by a general practitioner, who diagnosed migraine and prescribed a single 50 mg dose of 5-HT_1_ serotonin receptor agonist (sumatriptan). She had no history of ocular trauma and retinal or choroidal diseases. Previously, the patient has not been treated for ophthalmic reasons. She did not report chronic diseases and had no history of taking anticoagulants.

The patient was examined with slit lamp biomicroscopy with dilated fundus examination. On admission, the best corrected visual acuity (BVCA) according to the Snellen charts was hand motion in the RE and 0.6 in the left eye (LE), respectively. The intraocular pressure (IOP) measured with an applanation Goldmann tonometer was 60 mmHg in the RE and 30 mmHg in the LE. Examination of the anterior segment of the RE revealed a hard, heavily congested eyeball, edema of the proper corneal substance, shallowing of the anterior chamber, a non-reactive, wide, vertical-oval pupil and an opalescent lens. There were no abnormalities in the anterior segment of the LE. Dilated fundus examination revealed numerous drusen in the posterior pole and mid-periphery of both eyes.

The interview and physical examination led to the diagnosis of acute primary angle closure (APAC) in the RE. The patient received topical therapy including beta-blockers, carbonic anhydrase inhibitors, parasympathomimetic and steroids. She used general therapy of 500 mg of acetazolamide and 500 mL of 20% hyperosmotic Mannitol solution intravenously. After IOP decreasing in the RE to 10 mmHg and in the LE to 13 mmHg, she was referred for laser iridotomy in the RE and LE the following day.

The next day, BVCA of the RE was 0.2 and IOP was 8 mmHg. Fundus examination of the RE revealed diffuse retinal hemorrhages in the posterior pole.

Ocular decompression retinopathy (ODR) was first described in 1992 by Fechtner et al. in a patient after trabeculectomy with mitomycin C [1]. In the available literature it is presented as a rare complication associated with rapid decrease of IOP [2,3]. It is characterized by multiple retinal hemorrhages, which may occur in all retinal layers, mainly intra- and subretinally [3]. They are a consequence of both conservative and laser therapy or surgical procedures applied in cases of increased IOP [4,5,6]. Therefore, it is important to be aware of these possible retinal complications when pharmacological, laser or surgical treatment is conducted to reduce pressure values [7]. To the best of our knowledge, this is the first case that has presented unilateral ODR secondary to rapid IOP reduction. Overall, the development of retinal hemorrhages following conservative treatment of APAC was reported as exceedingly rare [8].

Clinical features of ODR in our patient were typical as in previously observed cases [1,2,3,4,5,6]. We observed hemorrhages in intra-retinal layers. Multiple intraretinal, small hemorrhages usually resolve themselves within a few weeks. We have monitored this process for a few months. Although, in the available literature, 26% of eyes with ODR showed changes in the optic nerve [3], abnormalities such as peripapillary and optic nerve head hemorrhages, optic disc hyperemia and disc edema were not found in our case.

The authors reported that a significant proportion of ODR cases (about 50%) occur in patients with a history of trabeculectomy [3]. Approximately 80% of patients were asymptomatic or mild in course [1,2,3]. The main symptoms reported by them included decreased vision, central scotoma and floaters. Mukkamala et al. mentioned that in 84% of cases, ODR occurs unilaterally [3]. Furthermore, in the rest of the patients (16%), the disease later affected the second eye. We did not find in the available literature that ODR can affect both eyes at the same time.

The exact pathophysiology of ODR is not well understood, but it is thought to be related to changes in retinal blood flow after IOP reduction. Two mechanisms in the pathophysiology of ODR were considered in the literature [3]. It was first suggested that there is a vascular mechanism. It was proposed that high IOP might contribute to impaired autoregulation of retinal capillaries, while a rapid IOP decreasing could cause leakage from these vessels, resulting in multiple hemorrhages [9]. The second mechanism (anatomical) was related to the forward displacement of the cribriform plate of the ethmoid bone. It was proposed that this mechanism causes inhibition of axoplasm transport in nerve fibers and compression of the central retinal vein (CRV), which leads to the formation of diffuse retinal hemorrhages. This mechanism may imitate CRV occlusion (CRVO) [3,10,11].

The patient qualified for urgent cataract surgery in the RE, which was performed within 3 days. On the next day after surgery, BVCA was 0.3 and IOP was 24 mmHg in the RE. Approximately two weeks later, the measurements were BVCA = 0.6 and IOP = 9 mmHg, respectively. At the same time, laser iridotomy in the LE was performed. Due to persistently elevated IOP in the LE (up to 27 mmHg), local hypotensive treatment was initiated. 

Five months later, examination of the RE revealed BVCA = 0.7 and IOP = 12 mmHg. Fundus eye photography showed absorbing retinal hemorrhages (Figure 1D). Due to the patient’s history, elevated IOP and increased lens thickness (5.15 mm) of the LE, the patient underwent cataract surgery in the LE and is currently taking a topical drug containing dorzolamide and thymolol twice daily to the LE.

The patient was monitored using multimodal imaging methods which included color fundus photography, optical coherence tomography (OCT) and OCT angiography (OCTA), as well as fluorescein angiography (FA). The examination was performed using SD-OCT (Spectralis, Heidelberg Engineering, Heidelberg, Germany), OCTA (Solix full-range OCT, Optovue Inc., Freemont, CA, USA) and ultra-widefield fluorescein angiography (UWF-FA) (California, Optos, Edinburgh, UK).

In our case, OCT B-scans demonstrated diffuse intraretinal hemorrhages in the RE and were performed in the same area on each follow-up visit (Figure 2). OCT was used to evaluate retinal thickness and morphology of ODR and to assess the integrity of the outer retina and the retinal pigment epithelium (RPE). This information of the retinal structure provided important insights into the pathophysiology of ODR in our patient. We did not observe photoreceptor pathology in the course of the disease.

In most of the previous reports, OCT scans of ODR were not included; therefore, a decrease in BVCA has been attributed to macular blot hemorrhage [12]. Mukkamala et al. found macular edema in 5% of the ODR cases [3]. Other authors reported that macular edema could be underdiagnosed by the lack of imaging methods in a higher percentage of eyes. They suggested that low BVCA should be associated with macular edema when hemorrhages are not observed in a disease picture [13].

Non-invasive OCTA and invasive UWF-FA were performed to assess the vascular structure of the posterior pole of the eye (Figure 3).

In the available literature, FA images demonstrated blocked fluorescence consistent with retinal hemorrhages. Most of the cases had normal retinal and choroidal vascular filling [7]. Similarly, UWF-FA of the RE in our patient showed areas of fluorescein flow blockage that correspond to hemorrhages in the posterior pole of the eye. It also revealed multiple foci of hyperfluorescence with the type of window defects corresponding to RPE damage. No delayed venous filling was found; thus, the thrombotic nature of the lesions was excluded. Furthermore, our study is the first using UWF imaging methods among others in monitoring ODR.

As in FA, vascular structure in OCTA was found to be normal with no evidence of impaired vascular perfusion or enlargement of the foveal avascular zone (FAZ), which was confirmed in previously published studies [14]. OCTA provided high quality angiograms, which showed normal retinal vascular configuration (as in FA) and was helpful in differentiating ODR from other retinal diseases, mainly from CRVO.

Based on the medical history, clinical picture and the course of treatment, the patient was diagnosed with ODR, which probably occurred due to a rapid decrease of IOP in the RE.

The ODR differential diagnosis includes, among others, CRVO often accompanied by macular edema. Compared to CRVO, ODR appears in patients who are typically younger, asymptomatic, and have a full return to pre-event fundus exam. With few exceptions, there is no venous dilatation in ODR, which can be observed in CRVO. It also includes Valsalva retinopathy (VR), which follows a sudden increase in pressure in the chest. In contrast to ODR, VR typically causes pre-retinal hemorrhages with a macular predilection. ODR hemorrhages tend to occur in all retinal layers. Other ocular conditions such as Terson’s syndrome occurring in sudden intracranial hemorrhage, as well as diabetic retinopathy, shaken baby syndrome, ocular ischemic syndrome and coagulopathies must be excluded in the diagnostics process [3,11].

In conclusion, ODR is a rare, usually self-limiting condition that is a result of a rapid decrease in IOP. In most cases, it is asymptomatic. In ODR, pharmacological, laser and surgical treatment methods are used to reduce IOP pressure values. Multimodal imaging methods, including color fundus photography, OCT, OCTA and FA, provide valuable insights into the pathophysiology of ODR and help in its differentiation from other retinal diseases. They usually show multiple intraretinal, small hemorrhages which resolve themselves within a few weeks. This is important in choosing the type of therapy; for example, if changes in the retinal blood flow contribute to the development of ODR, methods affecting vascular system may be more effective. Among others, OCT can be used to evaluate changes in retinal thickness and morphology and FA or OCTA to assess blood flow, which is helpful for clinicians to better understand the pathophysiology of ODR. To the best of our knowledge, this is the first report in which multimodal imaging techniques were used in monitoring ODR. It provides more detailed imaging information and some guidance to clinical ophthalmologists in managing the complications of APAC. Nowadays, these methods play a crucial role in illustrating retinal disorders and making therapeutic decisions in the course of the disease [15,16]. Additionally, our study is the first one summarizing imaging methods used in ODR evaluation and include UWF-FA images of this ocular condition. Future studies should focus on the use and monitoring of ODR by multimodal imaging methods to provide additional understanding of the course of the disease.

## Figures and Tables

**Figure 1:**
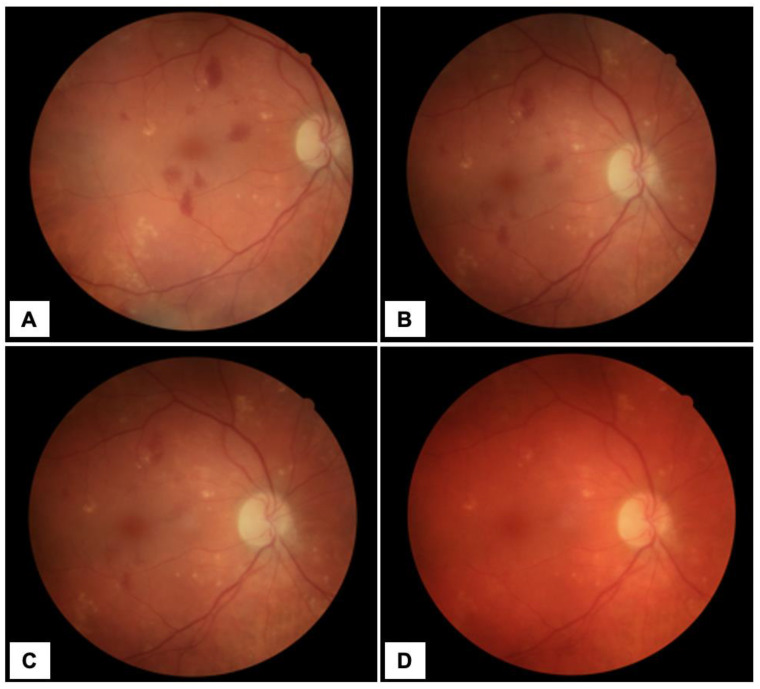
Color fundus photography of the patient’s right eye (RE) (Solix full-range OCT, Optovue Inc., Freemont, CA, USA) with ocular decompression retinopathy (ODR). Diffuse intraretinal hemorrhages are shown in the posterior pole and drusen in the posterior pole and mid-periphery of the RE. The photo was taken on the first day after admission to the hospital (**A**), after a month (**B**), 3 months (**C**) and 5 months of follow-up period (**D**).

**Figure 2:**
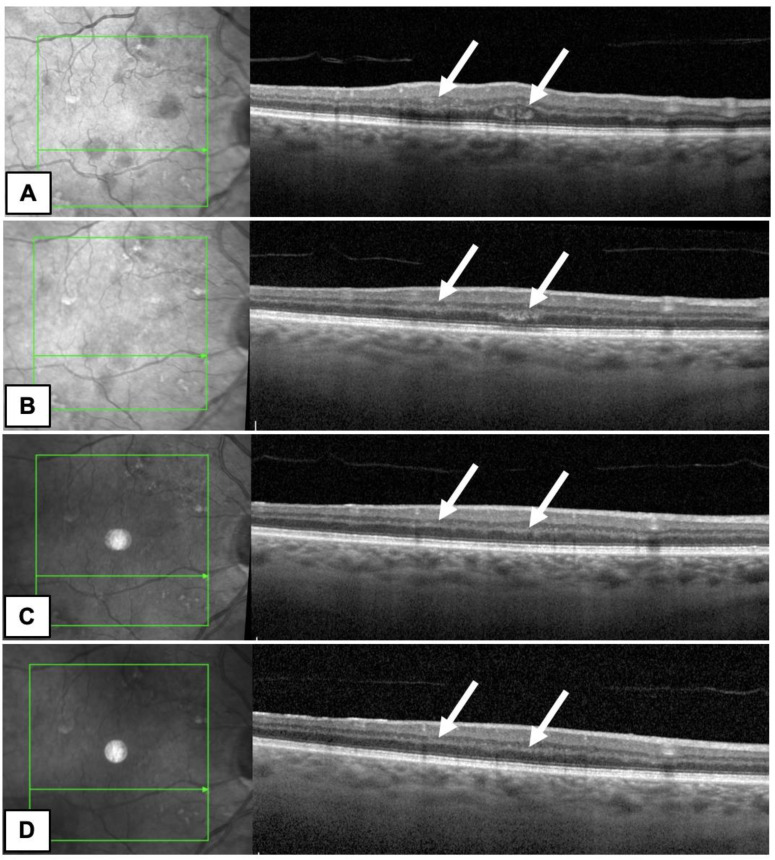
OCT B-scans of the patient’s RE showed intraretinal hemorrhages marked by white arrows ((**A**)—first day after diagnosis, (**B**)—after a month), which resolved during follow up ((**C**)—after 3 months, (**D**)—after 5 months).

**Figure 3:**
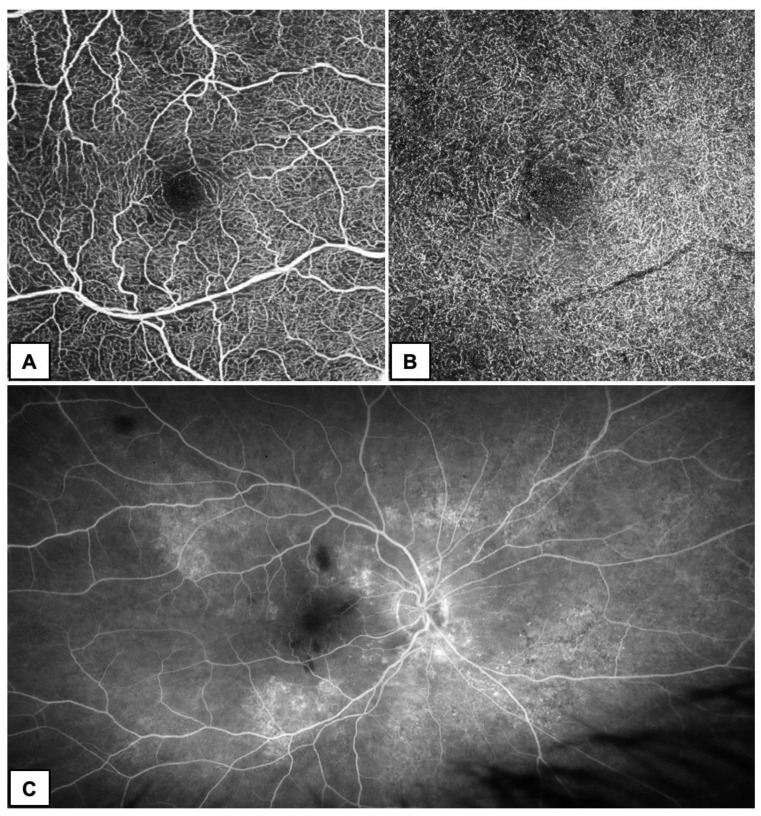
OCTA (**A**,**B**) and FA (**C**) of the patient’s RE. (**A**,**B**) OCTA the day after diagnosis revealed no abnormalities in both, superficial (**A**) and deep (**B**) capillary plexus except for a subtle capillary rarefraction around the foveal avascular zone (FAZ). (**C**) UWF-FA performed approximately one month after diagnosis showed fluorescein flow blockage corresponding to hemorrhages in the eye fundus. Vessel filling appeared normal.

## Data Availability

Data are available from the corresponding author upon reasonable request.
